# Efficacy and safety of Anluohuaxian in the treatment of patients with severe Coronavirus disease 2019- a multicenter, open label, randomized controlled study: a structured summary of a study protocol for a randomised controlled trial

**DOI:** 10.1186/s13063-020-04399-8

**Published:** 2020-06-08

**Authors:** Chi Zhang, Jiawen Li, Zhao Wu, He Wang, Chengli Que, Hong Zhao, Guiqiang Wang

**Affiliations:** 1 Department of Infectious Disease, Center for Liver Disease, Peking University First Hospital, No.8 Xishiku Street, Xicheng District, Beijing, China; 2 Department of Radiology, Peking University First Hospital, No. 8 Xishiku Street, Xicheng District, Beijing, 100034 China; 3 Department of Respiratory Disease, Peking University First Hospital, No. 8 Xishiku Street, Xicheng District, Beijing, 100034 China; 4 The Collaborative Innovation Center for Diagnosis and Treatment of Infectious Diseases, Zhejiang University, Hangzhou, Zhejiang China; 5 Peking University International Hospital, Beijing, China

**Keywords:** COVID-19, Randomised controlled trial, Protocol, Pulmonary fibrosis, Anluohuaxian

## Abstract

**Objectives:**

Patients with severe COVID-19 often suffer from significant pulmonary fibrosis. Although the pathogenesis of pulmonary fibrosis has not been fully explained, the signal pathways and cytokines involved are very similar to hepatic fibrosis. This has been successfully treated with the Anluohuaxian Pill, a proprietary Chinese medicine composed of a variety of Chinese herbal medicines. The aim of this study is to evaluate the efficacy and safety of Anluohuaxian in the treatment of pulmonary fibrosis in patients with severe COVID-19.

**Trial design:**

This is a prospective, multicenter, open, randomized controlled trial. The distribution ratio was 2:1, 500 cases in the experimental group and 250 cases in the control group.

**Participants:**

Minimum Age: 18 Years

Maximum Age: 80 Years

Sex: All

Gender Based: No

Accepts Healthy Volunteers: No

Inclusion Criteria:

1. Confirmed COVID-19, and the nucleic acid test of respiratory specimens such as sputum or nasopharyngeal swabs is negative twice after the treatment (sampling interval is at least 24 hours);
2. Negative nucleic acid test of respiratory specimens such as sputum or nasopharyngeal swabs during screening visits;
3. High-resolution CT of the lung (HRCT) indicates pulmonary fibrosis (thickness of lobular septum, honeycomb-like changes, with or without bronchial / pleural distraction);
4. Voluntarily participate in research and sign informed consent.

Exclusion Criteria:

1. Combined with severe heart, lung (diagnosed with interstitial lung disease, bronchial asthma, chronic obstructive pulmonary disease, etc.), liver and kidney disease or with endocrine, rheumatic, neurologic, malignant and other systemic diseases;
2. Have been diagnosed with connective tissue disease;
3. Pregnant or lactating women;
4. History of mental disorders, substance abuse or dependence;
5. Have used other anti-pulmonary fibrosis drugs in the past 14 days, such as nintedanib, pirfenidone, penicillamine, colchicine, tumor necrosis factor alpha blocker, imatinib, glucocorticoid hormones, morphomycodyl esters, azathioprine, cyclophosphamide, interferon-γ, and traditional Chinese medicine;
6. Researchers consider it inappropriate to participate in research;
7. Participating in other clinical research.

This mutli-centre RCT will be undertaken in 9 trial centres: The Second People's Hospital of Fuyang, Ezhou Central Hospital, Huoshenshan Hospital of Wuhan, Jinyintan Hospital of Wuhan, Tongji Hospital of Huazhong University of Science and Technology, West Hospital Union Hospital Huazhong University of Science and Technology, Wuhan Pulmonary Hospital, Zhongnan Hospital of Wuhan University, Wenzhou Medical University Affiliated First Hospital.

**Intervention and comparator:**

The research drug is Anluohuaxian Pill, which is provided by Senlong Pharmaceutical Co., Ltd. The basic therapeutic drugs for COVID-19 involved in the study include antiviral drugs. Brands can be selected according to the treatment routines of each research center to facilitate the improvement of treatment compliance.

**Main outcomes:**

Primary Outcome Measure:

1. Changes in high-resolution computer tomography of the lung

Changes in ground-glass shadows, interstitial or air nodules found on high-resolution computer tomography

[Time Frame: 3 months]

2. Change in 6-minute walking distance

[Time Frame: 3 months]

**Randomisation:**

In this study, the central randomization system (IWRS, an interactive network response system based on network) is used to randomise the groups. The subjects who met the entry criteria were randomly divided into the experimental group and the control group according to the proportion of 2:1. In this study, the block randomized grouping method is used, and the block length is 6. The random grouping program is set up by statistical and computer professionals in the randomization process.

**Blinding (masking):**

This is an open label trial. Trial participants, investigators, care givers, outcome assessors, and date analysts are not blinded to group assignment.

**Numbers to be randomised (sample size):**

750 patients are expected to be enrolled and the cases are allocated according to the ratio of 2 (Anluohuaxian combined with regular treatment group):1 (regular treatment group).

**Trial Status:**

Protocol version number 3.0, 10th April 2020. The recruitment has not yet started.

Actual Study Start Date: April 1, 2020

Estimated Primary Completion Date: June 1, 2020

Estimated Study Completion Date: December 1, 2020

**Trial registration:**

ClinicalTrials.gov ID: NCT04334265. Registered on 3 April 2020

**Full protocol:**

The full protocol is attached as an additional file, accessible from the Trials website (Additional file 1). In the interest in expediting dissemination of this material, the familiar formatting has been eliminated; this Letter serves as a summary of the key elements of the full protocol.

## Supplementary information

**Additional file 1.** Full study protocol.

## Data Availability

The datasets generated during and/or analyzed during the current study will be made available.

