# Supplementary material for: Efficacy and safety of Anluohuaxian in the treatment of patients with severe Coronavirus disease 2019- a multicenter, open label, randomized controlled study: a structured summary of a study protocol for a randomised controlled trial
Source: Trials. 2020 Jun 8;21:495. doi: 10.1186/s13063-020-04399-8 (PMC7276971; doi:10.1186/s13063-020-04399-8)
Supplement: Supplementary file 1 — Additional file 1. Full study protocol. [file 13063_2020_4399_MOESM1_ESM.docx]

**安络化纤丸治疗****COVID-19康复期患者疗效和安全性的多中心、开放、随机对照研究**

| **版 本 号：** | V 3.0 |
| --- | --- |
| **版本日期** | 2020年4月08日 |
| **项目申办单位：** | 北京大学第一医院 |
| **项目负责人：** | 王贵强 |
| **联系电话** | 13911405123 |
|  |  |
|  |  |
|  |  |
|  |  |

**保密声明**

本方案中所包含的所有信息的所有权归申办单位，仅提供给研究者、合作研究者、伦理委员会和监督管理部门等相关机构和人员审阅。在未得到申办单位书面批准情况下，除了在与可能参加本试验的受试者签署知情同意书时，向其做必要的解释外，严禁将任何信息告知与本试验无关的第三方。

**申办单位名称和地址：**

名 称： 北京大学第一医院（牵头负责单位）

地 址： 北京市西城区西什库大街8号

联 系 人： 王贵强

邮 编： 100034

电 话：

传 真：

手 机： 13911405123

E – mail： john131212@sina.com

**研究机构名称和地址：**

名 称：武汉肺科医院

联 系 人：陈先祥

电 话：18971570937

名 称： 武汉金银潭医院

联 系 人： 张定宇

手 机： 13507117929

名 称： 鄂州市中心医院

联 系 人： 喻军华

手 机： 13908688619

名 称：阜阳市第二人民医院

联 系 人：韩明锋

手 机：13955881280

武汉大学中南医院（雷神山）

联 系 人： 王行环

手 机：

石家庄市第五医院

联 系 人： 郑欢伟

手 机：

名 称：济南市传染病医院

联 系 人：张纵

手 机：

名 称：成都市公共卫生临床中心

联 系 人：曾义岚

手 机：

名 称：无锡市第五人民医院

联 系 人：邱源旺

手 机：

名 称：深圳市第三人民医院

联 系 人：刘映霞

手 机：

名 称：蚌埠医学院第一附属医院

联 系 人：李伟

手 机：

**研究人员**

研究负责人： 王贵强

研究设计者：王贵强，赵鸿

研究参加者： 王贵强，赵鸿，阙呈立，王鹤，陈先祥，张定宇，喻军华，朝明锋，王行环，郑欢伟，张纵，曾义岚，邱源旺，刘映霞，李伟。

**研究日期**

计划研究起始日期： 2020 年04月01日

计划研究完成日期： 2020 年12 月01日

**签名**

专题负责人： 王贵强 日期： 年 月 日

中心负责人： 日期： 年 月 日

委托方负责人： 日期： 年 月 日

**合规申明**

本研究遵照中国国家食品药品监督管理局颁布的《药物临床试验质量管理规范（GCP 现行版）》进行，并遵守世界医学大会赫尔辛基宣言（第 18 届世界医学协会联合大会，赫尔辛基，芬兰，1964 年 6 月）以及修订版。研究方案遵守地方法规， 与知情同意书的文档一起提交给伦理委员会审批批准后获得伦理批件。

伦理委员会的批准文件以书面的形式送交研究者，然后再由研究者将批准文件（原件或副本）提供给研究申办方。

筛选开始前，研究者对每个受试者解释了本次临床试验的目的、方法、益处和潜在的风险，并获得了临床试验的受试者签署的知情同意书。由临床试验的受试者本人（或法律监护人）签名和注明签署日期的知情同意书由研究者和受试者各自妥善保存一份。

在临床研究过程中，任何与临床研究安全性相关的问题，如临床研究方案的变更（本试验过程中未变更方案）以及临床研究中的严重不良事件，均应及时向伦理委员会报告。

临床研究结束时提交报告给伦理委员会。

**目录**

[方案摘要 6](#_Toc37234007)

[一、研究背景 8](#_Toc37234008)

[二、研究目的和治疗终点 9](#_Toc37234009)

[2.1研究目的 9](#_Toc37234010)

[2.2主要终点 10](#_Toc37234011)

[2.3次要终点 10](#_Toc37234012)

[三、研究方法 10](#_Toc37234013)

[3.1研究设计 10](#_Toc37234014)

[3.2研究的持续时间 10](#_Toc37234015)

[3.3研究对象 10](#_Toc37234016)

[3.4无效病例的剔除标准 11](#_Toc37234017)

[3.5退出标准 11](#_Toc37234018)

[3.6研究计划的终止标准 11](#_Toc37234019)

[3.7脱落标准 12](#_Toc37234020)

[3.8随机化 12](#_Toc37234021)

[3.9研究干预 12](#_Toc37234022)

[3.9.1研究药物 12](#_Toc37234023)

[3.9.2治疗方案 12](#_Toc37234024)

[3.9.3合并用药 12](#_Toc37234025)

[3.10疗效评估 13](#_Toc37234026)

[3.11安全性评估 13](#_Toc37234027)

[3.12访视计划 14](#_Toc37234028)

[3.12.1筛查/基线访视 17](#_Toc37234029)

[3.12.2 治疗1月时访视 17](#_Toc37234030)

[3.12.3治疗3月/停药3月时访视 18](#_Toc37234031)

[3.12.4 治疗2月/停药后1月、2月时访视 18](#_Toc37234032)

[四、数据管理和质量保证 19](#_Toc37234033)

[五、统计分析 19](#_Toc37234034)

[5.1样本量的确定 19](#_Toc37234035)

[5.2统计分析集 19](#_Toc37234036)

[5.3统计分析方法 20](#_Toc37234037)

[六、受试者保护：知情同意、收益风险、保密、利益冲突 21](#_Toc37234038)

[6.1伦理方面的考虑 21](#_Toc37234039)

[6.2试验方案的审批 21](#_Toc37234040)

[6.3知情同意过程和知情同意书文本 21](#_Toc37234041)

[6.4受试者获益和风险 21](#_Toc37234042)

[6.5保密声明 21](#_Toc37234043)

[七、不良事件、严重不良事件的评估及处理 22](#_Toc37234044)

[7.1不良事件观察 22](#_Toc37234045)

[7.2 AE分级 22](#_Toc37234046)

[7.3 AE与研究药物之间关系的判断 22](#_Toc37234047)

[7.4 AE的记录 23](#_Toc37234048)

[7.5 严重不良事件的报告和处理 23](#_Toc37234049)

[八、研究质量控制 24](#_Toc37234050)

[8.1 研究培训 24](#_Toc37234051)

[8.2 数据核查 24](#_Toc37234052)

[8.3 质量控制报告 25](#_Toc37234053)

[九、研究进度安排 25](#_Toc37234054)

[十、参考文献 26](#_Toc37234055)

[附录1：圣•乔治医院呼吸问题调查问卷(SGRQ) 28](#_Toc37234056)

[附录2：6分钟步行距离试验详细指南(ATS 2002) 35](#_Toc37234057)

[附录3：mMRC评分 45](#_Toc37234058)

# 方案摘要

| **研究名称** | 安络化纤丸治疗COVID-19康复期患者疗效和安全性的前瞻性、多中心、开放、随机对照研究 |
| --- | --- |
| **研究设计** | 前瞻性、多中心、开放、随机对照研究 |
| **试验目的** | 评估安络化纤丸预防/阻逆COVID-19患者肺纤维化进程、改善肺功能的疗效和安全性 |
| **研究分组及**  **治疗方案** | - 对照组（n=250）：予基础治疗（根据受试者的治疗需要，可服用化痰药、止咳药等，及除尼达尼布、吡非尼酮、具有抗肺纤维化作用的中（成）药之外的治疗药物） - 试验组（安络化纤丸治疗组，n=500）：基础治疗+安络化纤丸（6g，bid）。实验组接受安络化纤丸治疗3个月。   完成本研究所规定的3个月的治疗的两组受试者，将进入治疗后为期3个月的随访阶段。实验组停用安络化纤丸治疗。在后续为期3个月的随访期间，两组受试者均接受基础治疗。 |
| **计划中心数量** |  |
| **计划招募**  **受试者数量** | 750例 |
| **研究周期** | 8个月 |
| **入选标准** | （1）年龄18～80周岁，男性或女性；  （2）曾确诊为COVID-19，出院时连续两次痰或鼻咽拭子等呼吸道标本核酸检测阴性（采样时间至少间隔24小时），且符合以下两项之一：  2.1重型或危重型，或  2.2肺部高分辨率CT（HRCT）提示肺纤维化（小叶间隔增厚、蜂窝样改变，伴或不伴牵拉支气管扩张）  （3）筛选访视时痰或鼻咽拭子等呼吸道标本核酸检测阴性；  （4）自愿参与研究并签署知情同意书。 |
| **排除标准** | （1）合并严重的心、肺（患病前确诊过间质性肺病、支气管哮喘、慢性阻塞性肺病等）、肝、肾疾病或血液、内分泌、风湿免疫、神经、恶性肿瘤等系统性疾病，且未稳定控制者；  （2）曾确诊为结缔组织病；  （3）孕妇或哺乳期妇女；  （4）精神异常、药物滥用或依赖史；  （5）近14天使用过其他抗肺纤维化治疗药物，如尼达尼布、吡非尼酮、D青霉素胺、秋水仙碱、肿瘤坏死因子α阻滞剂、伊马替尼、糖皮质激素、吗替麦考酚酯、硫唑嘌呤、环磷酰胺、干扰素-γ）、具有抗肺纤维化作用的中（成）药治疗；  （6）研究者认为不适合参与研究；  （7）正在参与其他临床研究。 |
| **主要有效性**  **评价指标** | - 肺部HRCT改善（基线至治疗3月时） - 6分钟步行距离(6MWD)（基线至治疗3月时）的变化 |
| **次要评价**  **指标** | - 肺活量及弥散功能改善（基线至治疗3月时） - 复合生理指数的变化（CPI）（基线至治疗3月时） - 圣•乔治医院呼吸问题调查问卷（基线至治疗3月时）评分的变化 - 改良英国医学研究委员会呼吸困难量表(mMRC)评分的改善（基线至治疗3月时） |

# 一、研究背景

新冠肺炎自2019年底爆发以来，截止到2020年3月19日我国累计确诊81263人，死亡3250人（粗病死率4.0%）。国外累计确诊140894人，死亡5922人（粗死亡率4.2%）[1]

目前公布的两份新冠肺炎肺部病理学报告均提示有不同程度的肺纤维化。其中一例局部肺部病理为50岁男性病人，既往未提示基础肺部疾病，确诊后14天死亡，病理检查提示双侧弥漫性肺泡损伤，伴细胞黏液样纤维瘤渗出液[2]。另一例为尸检报告提示：85岁男性病人，既往未提示基础肺部疾病，从确诊到死亡仅15天，在肺切面灰白色黏稠液体溢出，并可见纤维条索[3]。

目前关于肺纤维化的原理还未完全阐述清楚，其可能机制是由肌成纤维细胞在慢性炎症反应中过度沉积细胞外基质（ECM）蛋白引起的瘢痕和组织硬化。多种有害刺激（包括毒素、传染性病原体、自身免疫反应和机械应激）能够诱导纤维化细胞反应。纤维化会影响身体的所有组织，如果不加以控制，会导致器官衰竭和死亡。目前对调节纤维化发生的关键信号转导通路的研究已经确定了潜在的治疗靶标，以阻止纤维化的进展并恢复细胞功能。为了响应组织损伤，源自多种来源的肌成纤维细胞（包括常驻成纤维细胞、间充质细胞、循环成纤维细胞以及其他细胞类型的转分化）可通过重塑细胞外环境来启动伤口愈合反应，以恢复组织完整性并促进实质细胞的替换。通常，当组织愈合时，这个促纤维化程序被关闭。然而，反复的损伤和修复会导致这一过程的失调，导致 ECM 蛋白在病理上的过度沉积，并伴随着肌成纤维细胞活性的上调，造成巨噬细胞和免疫细胞浸润的慢性炎症环境。在这种细胞环境中，细胞因子和生长因子被大量释放，包括转化生长因子-β（TGF-β）家族成员和 Wingless/Int-1（Wnt1），它们是纤维化过程的主要效应子[4]。TGF-β 和 Wnt1 结合其干细胞表面受体，并发起下游信号转导，最终分别导致 Smad2/3 和 CBP/β-Catenin 转录调节物的核易位。这导致靶基因表达上调，其功能进一步增强肌成纤维细胞分化和 ECM 蛋白（包括胶原、层粘连蛋白和纤维连接蛋白）的产生和分泌。随着过多 ECM 沉积的进行，基体的结构发生变化并变硬。细胞通过细胞表面整合素受体（激活 Hippo 信号转导通路及其主要下游效应子 YAP 和 TAZ）的机械传导感受 ECM 张力。在另一个前向循环中，激活的 YAP 和 TAZ 转位到胞核，促进包括 CTGF 和 PDGF 在内的促生长基因的上调，这些基因通过 PI3K/AKT/mTOR 通路促进肌成纤维细胞的增殖和活化。尽管细胞损伤和组织情况各异，但纤维化的机制都类似[5]。此外，除器官损伤外，纤维化还与肿瘤的发生发展有关，因为纤维化 ECM 可刺激细胞增殖并改变细胞极性，从而促进肿瘤发育和生长[6]。

安络化纤丸是由地黄、三七、水蛭、地龙等制成的黑褐色的浓缩丸，气微，味苦。目前应用于慢性乙型肝炎、乙肝后早、中期肝硬化等肝纤维化、肝硬化的治疗，具有软坚散结、凉血活血等作用。用法为口服，一次6g，一日2-3次。庄辉等对其机制进行了较深入的研究，27只雄性wistar大鼠随机分为对照组、肝纤维化模型组和肝纤维化模型+安络化纤丸治疗组，发现络化纤丸治疗3周后，肝细胞损害减轻，治疗组大鼠ALT水平较模型组显著降低（P=0.047），AST水平较模型组下降39．8％（P=0.053），安络化纤丸治疗6周后，治疗组大鼠肝纤维化程度较模型组有显著改善（P=0．002)。治疗组大鼠TGF -β基因mRNA和蛋白相对表达水平显著低于模型组(P=0．003)，其主要机制是抑制TGF -β的产生，从而发挥抗纤维化的效果[7]。庄辉等另一项研究纳入36 只Wistar 雄性大鼠随机分为对照组、模型组和治疗组。与模型组相比，安络化纤丸显著减轻了治疗组大鼠肝损伤，表现为大鼠一般状态、肝脏和牌脏形态、肝脏和脾脏指数、ALT 和AST 水平的改善。肝组织病理学诊断表明，治疗组大鼠肝纤维化程度较模型组显著改善(P = 0.015) 。治疗组大鼠月1 组织、中MMP-13 的mRNA 和蛋白相对表达水平显著高于模型组(mRNA: P = 0. 029 ;蛋白: P=0.016) ， 而MMP-2 和TIMP- 1/2 的mRNA 和蛋白相对表达水平显著低于模型组(mRNA: P = 0.048， P =0.023, P = 0.049; 蛋白: P = 0.03， P= 0.016，P = 0.003) 。安络化纤丸发挥抗肝纤维化作用另一个机制为通过改善肝功能、抑制肝星状细胞的激活、增强MMP-13 的表达、抑制MMP-2 和TIMP-1/ 2 的表达等实现[8]。综上，安络化纤在抗肝纤维化的机制与肺纤维化的发生发展均有TGF-β，MMP-13， MMP-2和TIMP- 1/2等参与。

王贵强等在安络化纤丸临床试验表明安络化纤丸联合恩替卡韦治疗可显著改善慢性乙型肝炎病毒感染者肝纤维化。最终共纳入219人（其中恩替卡韦单药组77人，恩替卡韦联合安络化纤丸组142人）的临床队列研究。治疗78 周后肝纤维化改善率为36.53%（80/219）、进展率为23.29%（51/219）。肝纤维化改善与基线纤维化程度和治疗方法相关（P < 0.05）。在安络化纤丸联合恩替卡韦治疗且基线肝纤维化评分（F）≥ 3 的患者中，肝纤维改善率（54.74%，52/95）显著高于仅接受恩替卡韦治疗者（33.33%，16/48），P = 0.016 ；联合治疗组肝纤维化进展比例（13.68%，13/95）在数值上低于单独治疗组（18.75%，9/48），P = 0.466。在基线F < 3 的患者中，联合治疗组肝纤维化改善和稳定的患者比例（68.08%，32/47）高于单独治疗组（51.72%，15/29）[9]。

鉴于安络化纤丸在肝纤维化中显示出有效阻逆纤维化作用，而肺纤维化和肝纤维化有者共同的作用机制，因此，安络化纤丸也应当具有阻逆COVID-19患者肺纤维化进程的作用。

# 二、研究目的和治疗终点

## 2.1研究目的

评估安络化纤丸预防/阻逆COVID-19患者肺纤维化进程、改善肺功能的疗效和安全性。

## 2.2主要终点

- 肺部HRCT改善（基线至治疗3月时）
- 6分钟步行距离(6MWT)（基线至治疗3月时）的变化

## 2.3次要终点

- 肺活量及弥散功能改善（基线至治疗3月时）
- 复合生理指数（CPI）的变化（基线至治疗3月时）
- 圣•乔治医院呼吸问题调查问卷（基线至治疗3月时）评分的变化
- 改良英国医学研究委员会呼吸困难量表(mMRC)评分的改善（基线至治疗3月时）

# 三、研究方法

## 3.1研究设计

（1）本研究采用前瞻性、多中心、开放、随机、对照研究设计，预计纳入750例受试者，按2:1比例进行随机，其中试验组（安络化纤丸治疗组）和对照组（基础治疗组）分别为500例和250例。

（2）两组受试者均不限制其COVID-19康复期相关的基础治疗，试验组在基础治疗以外加用安络化纤丸（6g，bid），治疗3个月，对照组仅接受基础治疗。治疗结束后随访观察3个月。

（3）当完成3个月治疗的安络化纤丸组受试者达到250例时，进行中期分析，并就分析结果决定是否扩大或者停止研究。

## 3.2研究的持续时间

本研究预期持续8个月，包括为期2个月的受试者招募期。

开始研究的日期：2020年4月1日。

最后一名受试者入组的日期：2020年6月1日。

研究结束的日期：2020年12月1日（最后1例合格受试者入选后满6个月）

数据库锁定时间：2021年3月1日。

## 3.3研究对象

本研究将入选18～80周岁的经治疗后符合出院标准的重型、危重型COVID-19康复期患者。受试者来自各研究中心收治的患者。各研究中心根据竞争入组原则入选符合本研究所定义的入选、排除标准的受试者。

**纳入标准：**

（1）年龄18～80周岁，男性或女性；

（2）曾确诊为COVID-19，出院时连续两次痰或鼻咽拭子等呼吸道标本核酸检测阴性（采样时间至少间隔24小时），且符合以下两项之一：

2.1重型或危重型，或

2.2肺部高分辨率CT（HRCT）提示肺纤维化（小叶间隔增厚、蜂窝样改变，伴或不伴牵拉支气管扩张）；

（3）筛选访视时痰或鼻咽拭子等呼吸道标本核酸检测阴性；

（4）自愿参与研究并签署知情同意书。

**排除标准：**

（1）合并严重的心、肺（患病前确诊过间质性肺病、支气管哮喘、慢性阻塞性肺病等）、肝、肾疾病或血液、内分泌、风湿免疫、神经、恶性肿瘤等系统性疾病，且未稳定控制者；

（2）曾确诊为结缔组织病；

（3）孕妇或哺乳期妇女；

（4）精神异常、药物滥用或依赖史；

（5）近14天使用过其他抗肺纤维化治疗药物，如尼达尼布、吡非尼酮、D青霉素胺、秋水仙碱、肿瘤坏死因子α阻滞剂、伊马替尼、糖皮质激素、吗替麦考酚酯、硫唑嘌呤、环磷酰胺、干扰素-γ、具有抗肺纤维化作用的中（成）药治疗；

（6）研究者认为不适合参与研究；

（7）正在参与其他临床研究。

## 3.4无效病例的剔除标准

（1）不符合入选标准的病例；

（2）符合排除标准的病例；

（3）未遵照方案要求服用治疗药物的病例；

（4）缺重要检查记录，或重要检查不符合要求的患者，包括：无疗效评估相关检查、无基本影像学检查。

## 3.5退出标准

（1）受试者发生严重不良事件，根据研究者判断必须中断研究；

（2）受试者怀孕；

（3）试验期间同时参与其他临床研究者；

（4）受试者自己要求退出；

（5）研究者认为需要中止试验。

退出病例应说明原因，并记录于电子病例报告表（eCRF）。有至少1次用药记录和安全性数据的受试者应纳入统计分析。

## 3.6研究计划的终止标准

（1）受试者撤回知情同意书；

（2）中期分析数据不支持进一步研究；

（3）受试者出现严重不良事件；

（4）治疗3个月后肺纤维化恶化者终止研究不再随访，以便受试者选择其他治疗。

## 3.7脱落标准

未完成临床试验方案的病例应视为脱落。包括病人自行退出（如依从性差，不愿意继续治疗等）及研究者令其退出（病情严重需合并应用其他药物而影响疗效判断者，重度不良事件需停药者）。脱落病例应说明原因，并将其最后一次主要疗效检测结果转接为最终结果纳入全分析集（FAS）进行统计分析。有一次用药记录均应纳入安全性分析集（SS），进行安全性分析。所有脱落病例应在eCRF中记录脱落原因。

## 3.8随机化

本研究采用中央随机化系统（基于网络的交互式网络应答系统，IWRS）实现随机分组。符合入排标准的受试者按2:1比例随机分为试验组和对照组。本研究采用区组随机化分组方法，区组长度为6。随机化过程由统计和计算机专业人员设定随机分组程序。

## 3.9研究干预

### 3.9.1研究药物

研究药物为安络化纤丸，由森隆药业有限公司提供。研究中涉及的COVID-19基础治疗药物包括抗病毒药物等，可依照各研究中心治疗常规选择品牌，便于提高治疗的依从性。

### 3.9.2治疗方案

签署知情同意书且符合入选排除标准的受试者将随机分为对照组（基础治疗组）和试验组（安络化纤丸+基础治疗组）。

基础治疗：根据受试者的治疗需要，可服用化痰药、止咳药等，及除尼达尼布、吡非尼酮、具有抗肺纤维化作用的中（成）药之外的治疗药物。

安络化纤丸：参考说明书，口服，一次6g，一日两次，治疗3个月。不良反应尚不明确。服药期间忌酒、辣椒。

完成本研究所规定的3个月的治疗的两组受试者，将进入治疗后为期3个月的随访阶段。实验组停用安络化纤丸治疗。在后续为期3个月的随访期间，两组受试者均接受基础治疗。对于治疗3个月后肺纤维化恶化者，终止研究不再随访以便患者应用其他治疗。

### 3.9.3合并用药

随机化后的3个月治疗期间，受试者不可合并使用尼达尼布、吡非尼酮、糖皮质激素、免疫抑制剂、具有抗肺纤维化作用的中（成）药。

## 3.10疗效评估

**主要疗效指标：**

- 肺部HRCT改善（基线至治疗3月时）
- 6分钟步行距离(6MWD)（基线至治疗3月时）的变化

**次要疗效指标：**

- - 肺活量及弥散功能改善（基线至治疗3月时）
  - 复合生理指数（CPI）的变化（基线至治疗3月时）
  - 圣•乔治医院呼吸问题调查问卷（基线至治疗3月时）评分的变化
  - 改良英国医学研究委员会呼吸困难量表(mMRC)评分的改善（基线至治疗3月时）

肺部HRCT改善的评估方法^10-14^：薄层CT表现分级为1-5，分级方法如下：1：正常。2：网格结构。3：牵拉性支扩。4：蜂窝结构。5:实变伴结构扭曲。由两位有胸部影像学诊断经验的影像科医师（10年及15年）观察员的评价影像资料后分级。将每一叶肺划分为上、中、下三个区域。气管隆突以上为上肺区，下肺静脉以下的区域为下肺区，两者之间为中肺区。在每一个肺区内，通过视觉估计来确定肺实质受累的百分比（精确到5%）。每个区域的得分乘以体积的百分比来得到最后评分。六个肺区得分的平均值为最终得分。得分最高为500分，最低得分为100分。我们将总评分命名为“CT纤维化评分”。在本研究中，影像科医师对受试者的随机分组及治疗情况持盲。

肺弥散功能检查按照中华医学会呼吸病学分会肺功能专业组编写的《肺功能检查指南》^15^中推荐的一口气呼吸法检查肺一氧化碳弥散功能（D_L_CO-sb）。

圣•乔治医院呼吸问题调查问卷详见附录1。

6分钟步行距离试验(6MWT)检查方法详见附录2。

改良英国医学研究委员会呼吸困难量表(mMRC)评分详见附录3。

复合生理指数（CPI）=91-(0.65×D_L_C0占预计值%)-(0.53×FVC占预计值%)+（0.34×FEV_1_占预计值%）。

## 3.11安全性评估

以生命体征、实验室指标（包括外周血细胞分类计数、肝肾功能）、不良事件（AE）、严重不良事件（SAE）作为安全性评估指标。不良事件根据美国卫生及公共服务部2017年公布的常⻅不良事件评价标准（CTCAE）5.0版进行观察和分级。

将用于评价安全性的终点如下：AEs的发生率；药物相关的AEs的发生率；死亡的发生率；SAEs的发生率；药物相关的SAE的发生率；导致治疗停止和提前退出研究的AEs的发生率。

## 3.12访视计划

根据当前的指南和队列研究所需要的信息，参照访视计划表内容进行受试者的访视，然而研究者也可以根据受试者的病情随时调整访视计划。

本研究访视阶段可分为两阶段。第一阶段为治疗期。入选受试者随机接受或不接受为期3个月的安络化纤丸治疗。完成本研究所规定的3个月治疗的受试者，将进入治疗后访视阶段，随访至停用安络化纤丸后3个月，随访期间禁止应用前述合并用药。对于治疗3个月后肺纤维化恶化者，终止研究不再随访以便患者接受其他治疗。

**访视计划表**

|  | **筛选/基线** | **治疗期** | | | **随访期** | | | **提前退出**  **/结束治疗** | **计划外访视** |
| --- | --- | --- | --- | --- | --- | --- | --- | --- | --- |
|  | **V0**  **筛选/基线期**  **（-14～0天）** | **V1**  **治疗1个月**  **±7天** | **V2**  **治疗2个月**  **±7天** | **V3**  **治疗3个月**  **±7天** | **V4**  **停药后1个月**  **±7天** | **V5**  **停药后2个月**  **±7天** | **V6**  **停药后3个月**  **±7天** |  |  |
| **采集基本病史** |  |  |  |  |  |  |  |  |  |
| 签署知情同意书 | X |  |  |  |  |  |  |  |  |
| 确定入选、排除标准 | X |  |  |  |  |  |  |  |  |
| 填写人口统计学资料 | X |  |  |  |  |  |  |  |  |
| COVID-19病史及治疗史 | X |  |  |  |  |  |  |  |  |
| 合并用药与合并治疗 | X |  |  | X |  |  | X | X | X |
| 体格检查 | X | X | X | X | X | X | X | X | X |
| 尿妊娠检查 | X |  |  |  |  |  |  |  |  |
| COVID-19病毒核酸检查（痰或咽拭子） | X |  |  |  |  |  |  |  | X |
| **有效性观察** |  |  |  |  |  |  |  |  |  |
| 血常规^1^ | X |  |  | X |  |  | X | X | X |
| T淋巴细胞亚群分析^2^ | X |  |  | X |  |  | X | X | X |
| 肝肾功能（ALT、AST、Cr、ALB、LDH） | X |  |  | X |  |  | X | X | X |
| 电解质（K、Na、Ca） | X |  |  | X |  |  | X | X | X |
| 纤维化四项 | X |  |  | X |  |  | X | X | X |
| KL-6 | X |  |  | X |  |  | X | X | X |
| 细胞因子水平^3^ | X |  |  | X |  |  | X | X | X |
| 尿液检查 | X |  |  | X |  |  | X | X | X |
| 血气分析 | X |  |  | X |  |  | X | X | X |
| 肺功能检查^4^ | X | X |  | X |  |  | X | X | X |
| 肺部HRCT检查 | X |  |  | X |  |  | X | X | X |
| 圣•乔治医院呼吸问题调查问卷（SGRQ） | X | X | X | X | X | X | X | X | X |
| 6分钟步行距离试验（6MWD） | X | X | X | X | X | X | X | X | X |
| 改良英国医学研究委员会呼吸困难量表(mMRC) | X | X | X | X | X | X | X | X | X |
| 复合生理指数（CPI） | X | X | X | X | X | X | X | X | X |
| **安全性观察** |  |  |  |  |  |  |  |  |  |
| 记录不良事件 | X | X | X | X | X | X | X | X | X |
| 记录严重不良事件 | X | X | X | X | X | X | X | X | X |
| 其它工作 |  |  |  |  |  |  |  |  |  |
| 随机分组 | X |  |  |  |  |  |  |  |  |
| 依从性评价 |  | X | X | X |  |  | X | X |  |
| 药物分发 | X | X | X |  |  |  |  |  | X |

备注：

1. 血常规检查：包括红细胞计数、血红蛋白、红细胞压积、白细胞计数、单核细胞计数及百分比、淋巴细胞计数及百分比、中性粒细胞计数及百分比、嗜酸性粒细胞计数及百分比和嗜碱性粒细胞计数及百分比。
2. T细胞亚群检测：包括CD3+占比和绝对值、CD4+占比和绝对值、CD8+占比和绝对值。
3. 细胞因子检测：包括TNF-α、IL-2、IL-4、IL-6、IFN-α、IFN-β、IFN-γ。
4. 采用流量型肺量计进行肺功能检查；采用一口气呼吸法检查肺一氧化碳弥散功能（筛选/基线访视时可不行肺功能检查）。

### 3.12.1筛查/基线访视

当获得受试者的书面知情同意后，研究者可对受试者进行筛查。各研究中心的研究者通过登陆EDC系统，录入所有进入筛查流程的潜在受试者信息。筛查/基线访视应在2周时间窗内完成。

筛查/基线访视时将收集以下资料：

（1）人口学信息：在筛查/基线访视时收集，内容包括年龄、性别。

（2）病史询问：筛查时应详细询问受试者病史，合并严重的心、肺（间质性病变、支气管哮喘、慢性阻塞性肺病等）、肝、肾疾病或血液、内分泌、风湿免疫、神经、恶性肿瘤等系统性疾病，且未稳定控制者将不被纳入。

（3）体格检查：内容包括体温、血压、心率

（4）COVID-19病毒核酸检查：痰或鼻咽拭子等呼吸道标本核酸检测阴性患者才考虑入组。

（5）血液检查：内容包括血常规、肝肾功能（谷丙转氨酶、谷草转氨酶、肌酐、白蛋白、乳酸脱氢酶）、电解质（钾钠钙离子）、纤维化四项、涎液化糖链抗原（KL-6）、淋巴细胞亚群分析、细胞因子水平。

（6）尿液检查：内容包括尿常规。

（7）尿妊娠检查

（8）血气分析：项目包括pH、PO_2_、PCO_2_。

（9）肺功能检查：采用流量型肺量计进行肺功能检查；采用一口气呼吸法检查肺一氧化碳弥散量

（10）影像学检查：进行肺部HRCT检查。

（11）圣•乔治医院呼吸问题调查问卷（SGRQ）

（12）6分钟步行距离试验（6MWD）

（13）改良英国医学研究委员会呼吸困难量表(mMRC)评分

（14）复合生理指数（CPI）

（15）合并用药：询问受试者合并用药情况，并记录于电子病例报告表中。

（16）不良事件：自患者签署知情同意书后，每次访视均应询问和记录不良事件，并按照CTCAE 5.0版进行分级。

### 3.12.2 治疗1月时访视

（1）体格检查：内容包括体温、血压、心率

（2）肺功能检查：采用流量型肺量计进行肺功能检查；采用一口气呼吸法检查肺一氧化碳弥散量

（3）圣•乔治医院呼吸问题调查问卷（SGRQ）

（4）6分钟步行距离试验（6MWD）

（5）改良英国医学研究委员会呼吸困难量表(mMRC)评分

（6）复合生理指数（CPI）

（7）合并用药：询问受试者合并用药情况，并记录于电子病例报告表中。

（8）不良事件：自患者签署知情同意书后，每次访视均应询问和记录不良事件，并按照CTCAE 5.0版进行分级。

### 3.12.3治疗3月/停药3月时访视

治疗3个月及停药后3个月访视时将收集以下资料：

（1）体格检查：内容包括体温、血压、心率

（2）血液检查：内容包括血常规、肝肾功能（谷丙转氨酶、谷草转氨酶、肌酐、白蛋白、乳酸脱氢酶）、电解质（钾钠钙离子）、纤维化四项、KL-6、淋巴细胞亚群分析、细胞因子水平

（3）尿液检查：内容包括尿常规

（4）血气分析

（5）肺功能检查：采用流量型肺量计进行肺功能检查；采用一口气呼吸法检查肺一氧化碳弥散量

（6）影像学检查：进行肺部HRCT检查。

（7）圣•乔治医院呼吸问题调查问卷（SGRQ）

（8）6分钟步行距离试验（6MWD）

（9）改良英国医学研究委员会呼吸困难量表(mMRC)评分

（10）复合生理指数（CPI）

（11）合并用药：询问受试者合并用药情况，并记录于电子病例报告表中。

（12）不良事件。

### 3.12.4 治疗2月/停药后1月、2月时访视

（1）体格检查：内容包括体温、血压、心率

（2）圣•乔治医院呼吸问题调查问卷（SGRQ）

（3）6分钟步行距离试验（6MWD）

（4）改良英国医学研究委员会呼吸困难量表(mMRC)评分

（5）复合生理指数（CPI）

（6）合并用药：询问受试者合并用药情况，并记录于电子病例报告表中。

（7）不良事件：自患者签署知情同意书后，每次访视均应询问和记录不良事件，并按照CTCAE 5.0版进行分级。

# 四、数据管理和质量保证

本试验采用电子病例报告表（eCRF）模式，在试验启动之前对试验参加人员进行相关培训。

（1）eCRF构建及审查：数据管理员根据 “研究病历”构建eCRF。构建完成后交研究者和申办者审查。一致通过后数据管理员根据研究者提供的信息创建帐号。

（2）EDC系统测试：对于构建好的eCRF进行试验前测试，确保无误并记录。

数据的录入：临床研究者应指定数据录入员，在受试者访视后，录入员应及时、准确地将研究病历中的数据录入到eCRF上，并输入电子签名（即帐号密码）。

监查员确认所有电子病例报告表填写的完整，并与原始资料一致，发现有错误的地方及时更正并电子签名。

（3）数据疑问及答疑：对eCRF中存在的疑问，监查员将随时在线提出疑问，研究者应尽快在线给予解答，修改错误数据，必要时监查员可以重复发出疑问。疑问及解答的交换应当采用疑问表形式，疑问表应保存备查。

（4）数据锁定及导出：在每一位受试者完成试验并经监查员审核无误后，由数据管理员进行数据逻辑核查和医学核查，若发现病例报告、严重不良事件，以及实验室数据存在疑问，数据管理员将产生对数据向研究者发出询问，在所有疑问均得到解决并确认建立的数据库正确后进行数据锁定，并完成数据管理审核报告。数据全部锁定后，由数据管理员导出数据库，交统计人员进行统计分析。

# 五、统计分析

## 5.1样本量的确定

预估总共纳入750例受试者，其中试验组500例，对照组250例。

## 5.2统计分析集

统计分析将用到3个分析集：全分析集（FAS）、符合方案集（PPS）和安全性分析集（SS）。同时采用FAS和PPS对所有疗效指标进行分析，如二者结果不一致，将进一步分析其原因。使用FAS进行分析时，对缺失数据将采用LOCF法（末次数据填补）进行数据填补。SS将用于安全性分析。

（1）全分析集（FAS）：包括所有符合入排标准进入研究，至少使用1次研究药物并有至少1次用药后疗效评价的对象。违背入排标准的病例将不包括在内。

（2）符合方案集（PPS）：包括所有符合入排标准进入研究，按照方案要求完成全部随访，没有重大方案违背，依从性良好（用药依从性≥80%）的对象。

（3）安全集（SS）：指所有进入研究，至少使用过1次研究药物并且有用药后安全性评价的病例。

## 5.3统计分析方法

**（1）一般原则**

除特别指出外，所有统计检验均为双侧检验，P<0.05即可认为所判断的差异有统计学意义。

- 定量数据：采用例数、算术均数、标准差、中位数和范围描述。
- 定性数据：采用频数、构成比或百分比描述。
- 统计检验：首先考虑用参数统计方法，如果数据分布与检验假设的要求相差较大，则用非参数统计方法。

数据库与统计分析: 数据库为epidata形式，用SAS 9.2统计软件进行统计分析。

**（2）病例特征**

- - 入组及完成情况：总结各中心入组及完成病例数，列出脱落病例的清单。
  - 一般信息的基线特征：基线定义为病例筛选期所获得的数据。对患者的人口学特征、病史等进行描述。

**（3）疗效评价**

- 疗效相关参数的基线评价: 与疗效评价相关的基线数据，定义为病例筛选期所获得的数据。采用FAS进行基线评价。分类变量采用CMH-χ2检验、Fisher’s精确概率法或等级数据的Wilcoxon秩和检验比较组间的差别。连续变量采用成组 t 检验或Wilcoxon秩和检验。分类疗效指标，如影像学评估资料的组间比较采用CMH-χ2检验或Logistic回归。

**（4）安全性评价**

- 对各治疗组的不良事件、严重不良事件分别统计发生例数，类别和严重程度；
- 采用CMH-χ2检验或Fisher’s精确概率法比较二组不良事件、不良反应的发生率；
- 采用成组t检验或Mann-Whitney法比较二组治疗后为连续变量的检查指标；
- 统计描述二组治疗后实验室指标正常、异常变化或异常加重的比例。

**（5）缺失数据处理**

对于受试者失访导致数据缺失，计划采用死亡或失访前所有可获得信息。主要结局分析时假设数据为随机缺失，并在敏感性分析中探讨失访是否与分析的结局相关。通过使用既往访视中得到的数据对失访进行预测来调查该问题的严重性，确定能够预测失访的变量将被纳入至预测模型中。此外，采用多重填补技术对主要和次要结局进行敏感性分析。

# 六、受试者保护：知情同意、收益风险、保密、利益冲突

## 6.1伦理方面的考虑

临床试验必须遵循赫尔辛基宣言和我国有关临床试验研究规范、法规进行。在试验开始之前，由临床试验负责单位的伦理委员会批准该试验方案后方可实施临床试验。

每一位受试者入选本研究前，研究医师有责任以书面文字形式，向其或其指定代表人完整、全面地介绍本研究的目的、程序和可能的风险。应让受试者知道他们有权随时退出本研究。入选前必须给每位受试者一份书面知情同意书，研究医师有责任让每位受试者在进入研究之前获得知情同意，知情同意书应作为临床试验文档保留备查。

## 6.2试验方案的审批

临床研究开始前向伦理委员会提供临床试验方案、详细的病人信息页和知情同意书的副本文件，以获得实施该临床研究的独立批准文件。如果后续方案有修正，将送交伦理委员会再次审核。

## 6.3知情同意过程和知情同意书文本

由研究者向受试者详细介绍本试验的背景、目的、步骤、获益、风险等情况，并解答受试者提出的试验相关的问题。

受试者充分了解本试验的背景、目的、步骤、风险及获益情况，对提出的问题得到答复后，在自愿参加本研究的情况下签署知情同意书。

知情同意书（另附）。

## 6.4受试者获益和风险

本研究将为所有受试者购买保险。

参与本次研究不能保证会从中获得临床益处。但是，从本次研究中获得的信息有利于将来应用安络化纤丸进行肺纤维化治疗的患者。本研究将为试验组受试者免费提供安络化纤丸治疗3个月。如研究结束（或中期结果分析）获得确切证据提示，安络化纤丸具有明显阻逆COVID-19患者肺纤维化进程、改善肺功能的作用，则将为对照组受试者提供3个月的安络化纤丸免费治疗。

研究药物安络化纤丸在肺纤维化患者中可能出现的不良反应尚不明确。鉴于安络化纤丸治疗肝硬化患者的安全性，预期其治疗因COVID-19导致肺纤维化的患者，不良反应风险可控。

## 6.5保密声明

本方案中所包含的所有信息的所有权归申办方所有，仅提供给研究者、合作研究者、伦理委员会和监督管理部门等相关机构和人员审阅。在未得到申办方书面批准情况下，除了在与可能参加本试验的受试者签署知情同意书时，向其做必要的解释外，严禁将任何信息告知与本试验无关的第三方。

# 七、不良事件、严重不良事件的评估及处理

## 7.1不良事件观察

不良事件（AE）定义：AE指临床研究对象在接受治疗方案后出现的任何不利的医学事件，但不一定与治疗有因果关系。

AE可以是和使用医学产品有时间上的联系的任何不愉快的或者和使用产品目的无关的体征（包括异常的实验室检查结果）、症状或疾病，不论是否认为与医学产品有关。

## 7.2 AE分级

AE按NCI《常见急性及亚急性毒性分级标准》（NCI-CTCAE 5.0）分为0-5级：

1级：轻度；无症状或轻微；仅为临床或诊断所见；无需治疗。

2级：中度；需要较小、局部或非侵入性治疗；与年龄相当的工具性日常生活活动（做饭、购买衣物、使用电话、理财等）受限。

3级：严重或者具重要医学意义但不会立即危及生命；导致住院或者延长住院时间；致残；自理性日常生活活动（洗澡、穿脱衣、吃饭、盥洗、服药等，并未卧床不起）受限。

4级：危及生命；需要紧急治疗。

5级：与AE相关的死亡。

如NCI毒性分级标准未列出的AE，可根据以下标准判断：

Ⅰ度（轻微）：有不舒服的感觉，但不影响正常的日常活动；

Ⅱ度（中度）：不舒服的程度达到足以减少或影响正常的日常活动；

Ⅲ度（严重）：不能工作或正常的日常活动；

Ⅳ度（致命）：致残或致死。

## 7.3 AE与研究药物之间关系的判断

按“肯定有关、很可能有关、可能有关、可能无关、无关”五级分类法对AE和研究药物之间可能存在的关联作出评估（见表1）。前三级判断为与药物相关。不良事件发生率计算时将三者合计作为分子，用于评价安全性的全部受试者例数作为分母。

表1：AE与治疗方案的关系判定标准

| **标准** | **肯定有关** | **很可能有关** | **可能有关** | **可能无关** | **无关** |
| --- | --- | --- | --- | --- | --- |
| 合理的时间顺序 | 是 | 是 | 是 | 是 | 否 |
| 已知的药物反应类型 | 是 | 是 | 是 | 否 | 否 |
| 去除原因可以改善 | 是 | 是 | 是或否 | 是或否 | 否 |
| 再次给药可重复出现 | 是 | ？ | ？ | ？ | 否 |
| 反应可能有另外解释 | 否 | 否 | 否 | 是 | 是 |

## 7.4 AE的记录

详细记录研究期间出现的各种AE名称、严重程度、出现时间、持续时间、处理措施、转归以及与治疗的关系等，并如实填写CRF。异常的实验室检查数据记录在CRF中，并重复该项检查每周至少一次，随访至恢复正常或研究结束。

## 7.5 严重不良事件的报告和处理

（1）严重不良事件的定义

严重不良事件（SAE）是指临床研究过程中发生的需要住院治疗或延长住院时间、伤残、影响工作能力、危及生命或死亡、导致先天畸形等医学事件。包括以下非预期医学事件：

- 导致死亡的事件；
- 危及生命的事件（定义为受试者在事件发生时有死亡危险）；
- 需要住院治疗或延长住院时间的事件；
- 可导致永久性或严重残疾/功能不全的事件；
- 先天异常或出生缺陷。

（2）药物过量

药物过量指受试者在24小时内（具体时间根据具体方案调整）加用研究药物，该剂量高于研究者医嘱规定的剂量。对于所有试验药物过量情况，不管是否与不良事件/严重不良事件相关，都应按严重不良事件报告。

（3）住院治疗

临床研究中导致住院治疗或住院时间延长的不良事件应视为严重不良事件。任何初次被医疗机构收住院（即使短于24小时）的情况均符合此标准。

住院不包括以下情况：

- 康复机构
- 疗养院
- 常规急诊室收治
- 当日手术（如门诊/当日/非卧床的手术）

与不良事件恶化无关的住院治疗或住院时间延长本身不是严重不良事件，例如：

- 因原有疾病入院，并没有新的不良事件的发生，也没有原有疾病的加重（如：为了检查研究前至今持续存在的实验室检查异常）；
- 管理原因的住院；
- 临床研究期间研究方案规定的住院；
- 与不良事件恶化无关的择期住院；
- 已预定的治疗或外科手术应在整个研究方案和/或受试者个人的基线资料中予以记录；
- 仅因为血液制品使用而入院。

诊断性或治疗性的侵入性（如手术）、非侵入性操作不应作为不良事件报告。但导致此项操作的疾病状况符合不良事件的定义时，应予以报告，如不良事件报告期间发生的急性阑尾炎应报告为不良事件，而因此进行的阑尾切除术应记录为该不良事件的治疗方法。

（4）SAE的报告程序

严重不良事件的报告应自受试者签署知情同意书开始，直至最后一次使用研究药物后的30个公历日（含第30天）。末次给药30天后发生的严重不良事件，除非怀疑与研究药品有关，一般不予报告。

试验期间，若发生严重不良事件必须在24小时内报告给临床监查员和主要研究者，同时填写《严重不良事件（SAE）报告表》，签名及注明日期，并以传真的形式立即上报申办单位、组长单位、研究单位伦理委员会、国家食品药品监督管理总局（CFDA）及研究者所在地区的（省或市）的食品药品监督管理局。

严重不良事件应详细记录症状、严重程度、发生时间、处理时间、采取措施、随访时间和方式以及转归情况。如果研究者认为某严重不良事件与试验药物无关，而与研究条件（例如终止原治疗，或试验过程中的合并症）潜在相关，则这种关系应在病历报告表的严重不良事件页的叙述部分详细说明。如果某种正在发生的严重不良事件的强度或其与受试药物的关系发生改变，应立即将严重不良事件随访报告送交申办者。所有的严重不良事件均应随访至恢复或稳定。

# 八、研究质量控制

质量控制是本项研究的关键。质量控制体现在受试者筛查和招募、基线数据获取、随访数据获取、结局定义和评估等各个研究环节。通过设置质量控制委员会，制定质量控制方案，进行质量控制评估并将结果反馈至各研究中心或研究其他部门，以维持和提高整个试验过程研究数据的质量。

## 8.1 研究培训

人员培训和操作流程的预先检验对于操作流程的标准化以及保证数据质量至关重要。基于研究方案对研究人员进行详细培训。研究方案是进行标准化研究流程的一个重要规范和参考。要求所有研究人员参加研究集中性或区域性培训课程。除外研究启动前集中培训及研究过程中定期开展研究培训外，研究质量控制或质量稽查人员可定期或按需前往研究中心对研究人员进行现场培训。

## 8.2 数据核查

研究负责单位将负责数据核查，包括对丢失数据、不真实值的核对，对不一致数据的交叉核对，以及对数据的抽查核对。所有数据问题将及时返还给数据录入/转换员以便及时处理。数据核查人员在网上生成数据质询报告，这些报告总结了研究数据质询的数目和类型。数据录入/转换员和研究中心的研究人员负责及时复审以及解决数据问题。

## 8.3 质量控制报告

质量控制委员会使用质量评估指标记录数据质量并给各个研究中心提供反馈，进一步在质量控制报告中持续跟踪这些质量评估指标。质量控制委员会将生成质量控制报告分发至各研究中心，并进行定期电话联系或研究中心随访讨论，确保各研究中心研究质量。

# 九、研究进度安排

20200315-20200401:完成方案设计、伦理审批、药品准备、CRO公司等相关工作；

20200401-20200601:完成病例筛选入组；

20200601-20200901:完成试验；

20200901-20201201:完成患者随访；

20201201-20210201:关闭研究、整理数据、撰写研究报告。

# 十、参考文献

1.http://2019ncov.chinacdc.cn/2019-nCoV/ (accessed March 19, 2020).

2.Zhe Xu, Lei Shi, Yijin Wang, et al., Pathological findings of COVID-19 associated with acute respiratory distress syndrome. The Lancet Respiratory Medicine, 2020.

3.刘茜，王荣帅，屈国强，等.新型冠状病毒肺炎死亡尸体系统解剖大体观察报告 法医学杂志, 2020,36(1): 21-23

4.Nanthakumar CB, Hatley RJ, Lemma S, et al., Dissecting fibrosis: therapeutic insights from the small-molecule toolbox. Nature reviews. Drug discovery, 2015. 14(10): p. 693-720.

5.Richeldi L, Collard HR and Jones MG, Idiopathic pulmonary fibrosis. Lancet (London, England), 2017. 389(10082): p. 1941-1952.

6.Mora AL, Rojas M, Pardo A, et al., Emerging therapies for idiopathic pulmonary fibrosis, a progressive age-related disease. Nature reviews. Drug discovery, 2017. 16(11): p. 755-772.

7.卢玮, 高玉华, 王珍子, et al., 安络化纤丸对肝纤维化大鼠转化生长因子β1及相应信号通路的影响. 中华肝脏病杂志, 2017. 25(4): p. 257-262.

8.王林, 卢玮, 高玉华, et al., 安络化纤丸对肝纤维化大鼠肝组织基质金属蛋白酶及其抑制物表达的影响. 中华肝脏病杂志, 2019. 27(4): p. 267-273.

9.苗亮, 杨婉娜, 董晓琴, et al., 安络化纤丸联合恩替卡韦治疗可显著提高慢性乙型肝炎病毒感染者肝纤维化的改善率. 中华肝脏病杂志, 2019. 27(7): p. 521-526.

10.Flaherty KR, Toews GB, Travis WD, et al. Clinical significance of histologicalclassification of idiopathic interstitial pneumonia. Eur Respir J 2002, 19:275–283.

11.Nicholson AG, Colby TV, du Bois RM, et al. The prognostic significance of the histologic pattern of interstitial pneumonia in patients presenting with the clinical entity of cryptogenic fibrosing alveolitis. Am J Respir Crit Care Med 2000, 162:2213–2217.

12.Rudd RM, Prescott RJ, Chalmers JC, et al. British Thoracic Society Study on cryptogenic fibrosing alveolitis: Response to treatment and survival. Thorax 2007, 62:62–66.

13.Oda K, Ishimoto H, Yatera K, et al. High-resolution CT scoring system-based grading scale predicts the clinical outcomes in patients with idiopathic pulmonary fibrosis. *Respir Res*. 2014;15(1):10. Published 2014 Jan 30.

14.Salaffi F, Carotti M, Tardella M, et al. Computed tomography assessment of evolution of interstitial lung disease in systemic sclerosis: Comparison of two scoring systems [published online ahead of print, 2020 Feb 20]. *Eur J Intern Med*. 2020;S0953-6205(20)30052-2. doi:10.1016/j.ejim.2020.02.009

15.中华医学会呼吸病学分会肺功能专业组.肺功能检查指南——肺弥散功能检查.中华结核和呼吸杂志.2015;38(3):164-169.

# 附录1：圣•乔治医院呼吸问题调查问卷(SGRQ)

| **圣•乔治医院呼吸问题调查问卷(SGRQ)** |
| --- |

*这份问卷是用来帮助我们更进一步了解你的呼吸问题是如何正在困扰你的，以及它是如何影响你的生活的。我们通过它发现疾病在哪一方面对你的影响最大,但这不是医生或护士所认为的那些问题。*

*请仔细阅读下列指导性语句，若有不明白之处请提问。不要花费太长的时间来决定你的答案。*

| *在完成余下的问卷前，请选择一个能体现你目前健康状况的描述并在小框中*  *打 “√”：* | 很好  🞎 (1) | 好  🞎 (2) | 一般  🞎 (3) | 不好  🞎 (4) | 很差  🞎 (5) |
| --- | --- | --- | --- | --- | --- |

第一部分

*关于在过去3个月内有关你的呼吸困难问题，每个问题只选择一个答案。*

|  | 一周中的绝大部分时间 | 一周中有几天 | 一个月中的几天 | 仅在有肺部感染时 | 没有 |
| --- | --- | --- | --- | --- | --- |
| 1. 在过去3个月内，咳嗽： | 🞎 (4) | 🞎 (3) | 🞎 (2) | 🞎 (1) | 🞎 (0) |
| 2. 在过去3个月内，我咳过痰： | 🞎 | 🞎 | 🞎 | 🞎 | 🞎 |
| 1. 在过去3个月内，我出现呼吸急促： | 🞎 | 🞎 | 🞎 | 🞎 | 🞎 |
| 4. 在过去3个月内内，我出现喘息发作： | 🞎 | 🞎 | 🞎 | 🞎 | 🞎 |
| 1. 在过去3个月内，你有过几次严重的或极不舒服的呼吸困难发作？ | 超过3次  🞎 (4) | 3次发作  🞎 (3) | 2 次发作  🞎 (2) | 1次发作  🞎 (1) | 没有发作  🞎 (0) |
| 6. 最严重的一次呼吸困难发作持续多长时间（若没有严重发作则跳过此题直接回答第7题）？ | 一周或更长时间  🞎 (3) | 3天或更长时间  🞎 (2) | 1至2天  🞎 (1) | 不超过1天  🞎 (0) |  |
| 7. 在过去3个月内，平均每周有几天是正常的（几乎没有呼吸困难）？. | 没有一天正常  🞎 (4) | 1到2天正常  🞎 (3) | 3 至 4天正常  🞎 (2) | 几乎每一天都是正常的  🞎 (1) | 每一天都正常  🞎 (0) |
| 1. 如果你有喘息，是否在清晨醒来时加重？ | 否  🞎 (0) | 是  🞎 (1) |  |  |  |

**第二部分**

| 一． | *你将如何描述你目前的呼吸困难？*  *请选择一个合适的框并打 “*√*”*： | 呼吸困难使我受到最严重的困扰  🞎 (3) | 呼吸困难使我受到相当多的困扰  🞎 (2) | | 呼吸困难使我受到一些困扰  🞎 (1) | | | 呼吸困难没有使我受到困扰  🞎 (0) | |
| --- | --- | --- | --- | --- | --- | --- | --- | --- | --- |
|  |  |  |  | |  | | |  | |
|  | *如果你曾经有过工作，请从中选择一项：* | 我的呼吸问题使我完全终止工作  🞎 (2) | | 我的呼吸问题影响我的工作或使我变换工作  🞎 (1) | | | 我的呼吸问题不影响我的工作  🞎 (0) | | |
|  |  | | | | | | | | |
|  |  | | | | | | | | |
| 二． | *下面问题是关于这些天来下列哪些活动经常让你觉得喘不过气来。*  *对每一个问题，请根据你的实际情况选择 “是 ” 或 “否”并在 框中打 “*√ *”：* | | | | | | | | |
|  |  | | | | |  | | |  |
|  | 静坐或静躺 | | | | | 是  🞎 (1) | | | 否  🞎 (0) |
|  | 洗漱或穿衣 | | | | | 🞎 | | | 🞎 |
|  | 在室内走动 | | | | | 🞎 | | | 🞎 |
|  | 在户外平地上走动 | | | | | 🞎 | | | 🞎 |
|  | 走楼梯上一层楼 | | | | | 🞎 | | | 🞎 |
|  | 爬坡 | | | | | 🞎 | | | 🞎 |
|  | 运动性体育活动或运动性游戏 | | | | | 🞎 | | | 🞎 |
|  |  | | | | | | | | |
|  |  | | | | | | | | |
| 三． | *下列问题是关于这些天来你的咳嗽及气喘问题。*  *对每一个问题，请根据你的实际情况选择 “是 ” 或 “否”并在 框中打 “*√*”：* | | | | | | | | |
|  |  | | | | | | | | |
|  | 我的咳嗽使我感到痛苦 | | | | | 是  🞎 (1) | | | 否  🞎 (0) |
|  | 我的咳嗽让我感到疲倦 | | | | | 🞎 | | | 🞎 |
|  | 谈话时会感到喘不过气来 | | | | | 🞎 | | | 🞎 |
|  | 我弯腰时觉得喘不过气来 | | | | | 🞎 | | | 🞎 |
|  | 我的咳嗽或呼吸影响我的睡眠 | | | | | 🞎 | | | 🞎 |
|  | 我很容易感到疲惫不堪 | | | | | 🞎 | | | 🞎 |

| 四． | *下列问题是关于这些天来你的呼吸困难可能对你其它方面的影响。*  *对每一个问题，请根据你的实际情况选择 “是 ” 或 “否”并在 框中打 “*√*”：* | | |
| --- | --- | --- | --- |
|  |  | | |
|  | 我的咳嗽及呼吸困难让我在他人面前感到难堪 | 是  🞎 (1) | 否  🞎 (0) |
|  | 我的呼吸问题让我的家人、朋友及邻居感到烦恼 | 🞎 | 🞎 |
|  | 当我喘不上气来时我感到害怕或惊恐 | 🞎 | 🞎 |
|  | 我觉得我无法控制我的呼吸问题 | 🞎 | 🞎 |
|  | 我不指望我的呼吸问题能好转 | 🞎 | 🞎 |
|  | 我的呼吸问题使我变得虚弱或致残 | 🞎 | 🞎 |
|  | 体育运动对我来说是不安全的 | 🞎 | 🞎 |
|  | 做任何事情做起来都很吃力 | 🞎 | 🞎 |
|  |  | | |
|  |  | | |
| 五． | *下列问题是关于你的治疗情况，若没有经过治疗请跳过这些问题直接回答第六大题。*  *对每一个问题，请根据你的实际情况选择 “是 ” 或 “否”并在 框中打* ”√”*：* | | |
|  |  |  |  |
|  | 我的治疗对我来说没多大帮助 | 是  🞎 (1) | 否  🞎 (0) |
|  | 在他人面前用药让我感到难堪 | 🞎 | 🞎 |
|  | 我的治疗对我有不良的药物副作用 | 🞎 | 🞎 |
|  | 我的治疗对我的生活干扰很大 | 🞎 | 🞎 |
|  |  | | |

| 六． | *下列问题是关于你的呼吸困难如何对你的活动可能造成的影响。*  *对于每一个问题，如果其中的一个或更多的项目因你的呼吸困难而受影响，请选择 “是 ”，否则选择 “否”并在 框中打 “*√*”：* | | |
| --- | --- | --- | --- |
|  |  |  |  |
|  | 我花很长时间进行洗脸涮牙或穿衣 | 是  🞎 (1) | 否  🞎 (0) |
|  | 我无法洗澡或淋浴，或需要花很长时间 | 🞎 | 🞎 |
|  | 我走得比别人慢，或需要停下来歇歇 | 🞎 | 🞎 |
|  | 诸如家务事要花长时间来做，或需要停下来歇歇 | 🞎 | 🞎 |
|  | 上一层楼梯时，我不得不慢慢走或停下来歇歇 | 🞎 | 🞎 |
|  | 若赶时间或快走，我不得不停下来休息或放慢速度 | 🞎 | 🞎 |
|  | 我的呼吸问题使我在进行诸如上坡、提东西上楼、跳舞、练气功或做操等活动时感到很困难 | 🞎 | 🞎 |
|  | 我的呼吸问题使我在进行诸如搬运重物、在花园中挖土、慢跑或快走(8公里/小时)、舞剑或游泳等活动时感到很困难 | 🞎 | 🞎 |
|  | 我的呼吸问题使我在进行诸如重体力活、跑步、骑自行车、快速游泳或进行剧烈体育活动时感到很困难 | 🞎 | 🞎 |
|  |  | | |

| 七． | *我们想知道你的呼吸问题通常是如何影响你的日常生活。*  *请选择是或否：*  *（记住必须是因为你的呼吸问题导致你不能做的这些活动才选择”是”）* | | | | | | |
| --- | --- | --- | --- | --- | --- | --- | --- |
|  |  | | | |  | |  |
|  | 我不能进行体育运动或做运动性游戏 | | | | 是  🞎 (1) | | 否  🞎 (0) |
|  | 我不能外出娱乐或消遣 | | | | 🞎 | | 🞎 |
|  | 我不能外出购物 | | | | 🞎 | | 🞎 |
|  | 我不能做家务 | | | | 🞎 | | 🞎 |
|  | 我不能走得离床或椅子太远 | | | | 🞎 | | 🞎 |
|  |  | | | | | | |
|  | *以下是一些由于你的呼吸问题而(或)无法进行的其它活动项目。（你不必选择是与否，它们只是提醒你气喘对你的影响。）*  ⚫ 散步或遛狗  ⚫ 在家干活  ⚫ 性生活  ⚫ 去教堂、酒吧或娱乐休闲场所  ⚫ 在天气不好时外出或进有烟味的房间  ⚫ 探亲访友或与孩子玩耍  *请在下面写下由于你的呼吸问题而无法进行的其它重要活动：*  ________________________________________________________________________________________________________________________________________________________________________________________________________________________________________________________________________________________________ | | | | | | |
|  | *现在，请选择一项最能反映*  *你的呼吸问题对你的影响的项目并在框中打 “√”：* | 不妨碍我做任何我想做的事情  🞎 (0) | 有1~2件我想做的事情会受到妨碍  🞎 (1) | 我想做的大多数事情都受到妨碍  🞎 (2) | | 所有我想做的事情都受到妨碍  🞎 (3) | |

# 附录2：6分钟步行距离试验详细指南(ATS 2002)

**6分钟步行距离试验详细指南(ATS 2002)**

**目的和范围**

该指南指导六分钟步行距离试验（6MWT）的临床应用。它综述了适应症、影响结果的各种因素、给出了具体的操作方案和安全测试方法，描述了患者的正确准备程序，并且提供了试验结果的临床解释。这些建议并非要限制其他方案在研究工作中的使用，也不就临床运动试验的话题展开讨论。

美国胸科学会关于肺功能测试的各种指南都是共识会议的结果。草稿由P.L.E.和R.J.Z.两位委员准备，参考了自1970年到2001年Medline的综合搜索文献结果，并且增加了其他委员的建议。草稿对于工作组提出的问题都做了相应的修改。指南尽量延用以前发表的方法并且对每一具体建议都提供了理论依据。最终的建议为委员会的一致意见。委员会建议该指南应该被考察5年，同时鼓励对有争议的地方进行更深入地研究。

**背景**

对于功能代偿能力的客观评价有几种不同的形式。有些对于操作过程中涉及的所有系统都提供了非常完整的评价标准（技术含量高），而其他的则仅提供了基本的信息，技术含量低但容易操作。具体形式的选择基于临床需要解决的问题和可以获得的资源。最流行的临床运动试验按复杂程度由低到高依次为爬楼、6MWT、往返步行试验、运动性哮喘检测、心脏负荷试验(如Bruce方案)和心肺运动试验。其他专业组织已经发表了心脏负荷试验的标准。

功能代偿能力的评价通常的做法仅仅是询问患者如下问题：“您能爬几层楼或您能走几个街区？”然而，患者记忆力不同可能高估或低估他们实际的功能代偿能力。客观的测量通常要好于自我报告。在20世纪60年代早期，Balke提出了一个简单的评价功能代偿能力的方法，即测量在规定的时间内的步行距离。然后发展出了测定健康人体能的12分钟场地步行试验。步行试验也适用于评价慢性支气管炎患者的功能受损情况。能够容纳并让呼吸疾病患者步行12分钟操作起来困难较大，而6分钟步行与12分钟步行效果相同。最近一篇关于功能性步行试验的综述得出结论“与其他步行试验相比，6MWT易于管理、耐受性更好、并且更能反映日常活动能力”。

6MWT简单易行，仅需要100英尺的走廊而不需运动器械或对技术员进行高级培训。步行是除了重病患者以外所有人都要进行的一种活动。该试验测定患者6分钟内在平坦、硬地上快速步行的距离。它评价了运动过程中所有系统全面完整的反应，包括肺、心血管系统、体循环、外周循环、血液、神经肌肉单元和肌肉代谢。它没有象最大量心肺功能运动测试那样提供关于运动中牵涉到的不同器官和系统功能的详细信息或运动受限的机制。自定速度的六分钟步行试验评价次大量功能代偿能力水平。多数患者在六分钟步行距离试验中不能达到最大运动量，他们选择自己的运动强度并且允许试验过程中停止行走和休息。然而，因为日常生活中多数活动需要在次大运动量水平完成，所以六分钟步行距离试验最好能反映能完成日常体力活动的功能代偿能力水平。

**适应症和限制**

6MWT主要适用于测量中到重度心脏或肺疾病患者对于医疗干预的反应，也可用于评价患者功能状态或预测发病率和死亡率（表2为适应症列表）。事实上在使用6MWT后，研究者们认为其对于测定这些疾病患者的功能代偿能力或给予干预后功能代偿能力的改变的临床作用（或最好的试验）并未完全得到证实。6MWT在各种临床情况的用途需要进一步研究。

**表2.六分钟步行距离试验的适应症**

| **治疗前和治疗后的比较** |
| --- |
| 肺移植 |
| 肺切除 |
| 肺减容术 |
| 肺的康复 |
| COPD |
| 肺循环高压 |
| 心力衰竭 |
| **评价功能状态（单一测量）** |
| COPD |
| 肺囊性纤维化 |
| 心力衰竭 |
| 周围血管疾病 |
| 纤维肌痛 |
| 老年患者 |
| **预测发病率和死亡率** |
| 心力衰竭 |
| COPD |
| 特发性肺动脉高压 |

正式的心肺运动试验能全面的评价对于运动的反应、客观的检测功能代偿能力和受损情况、测定延长运动所需的适当的运动强度、量化限制运动的因素并且定义基础的病理生理学机制如不同器官系统在运动中的作用。6MWT没有测定峰值氧耗量，也没有明确活动后呼吸困难产生的原因以及活动受限的原因或机制。6MWT 所提供的信息应作为心肺运动试验的补充而不是替代。尽管这两种功能试验存在许多差异，但是也有很好的相关性报道。例如，在肺疾病晚期患者的6MWD和峰值氧耗量显著相关(r=0.73)。
 在一些临床情况下，6MWT比峰值氧耗量能更好的评价患者日常生活能力。例如6MWD与正式的生活质量测试有更好的相关性(37)，干预治疗后6MWT的变化与患者呼吸困难的改善相关。在COPD患者中6MWT（变异系数约为8%）比FEV1.0重复性更好。与6MWT相比,功能状态的问卷测试结果短时间内变异性更大(22-33%)。

往返步行试验与6MWT相似，但它使用录音带的声音信号调节患者在10米的路程上来回步行的速度。步行速度每分钟都会提高，当患者不能在要求的时间内到达折返地点时试验就结束了。这种运动过程与症状限制的最大渐增运动负荷试验相似。往返步行试验的优点是与6MWT相比，它与峰值氧耗量的相关性更好。其缺点包括有效性差、应用范围小和具有更多潜在的心血管问题。

**禁忌症**

6MWT的绝对禁忌症包括1月内有不稳定性心绞痛或心肌梗死。相对禁忌症包括静息状态心率超过120次/分，收缩压超过180mmHg，舒张压超过100mmHg。

具有上述任何情况的患者都应该告知申请或指导检查的医师，以便于他们临床评价和决定是否进行该检查。6个月内的心电图结果也应该在检查前进行回顾。稳定的劳力性心绞痛不是6MWT的绝对禁忌症，但患者应在使用治疗心绞痛药物后进行试验，并且应备好急救用硝酸酯类药。

**理论依据**

具有上述危险因素的患者在试验过程中发生心律失常或心血管病的危险增加。然而，试验时患者可以根据自身情况来决定运动强度，并且已有数以千计的老年人、心衰或心肌病患者在无心电监测的情况下进行了该试验而没有发生严重不良事件。上述禁忌症是根据研究者对于6MWT的安全性的一般看法和出于谨慎的态度而制定。如果这些患者进行6MWT，没有人知道是否会发生不良事件，因此它们被列为相对禁忌症。

**安全问题**

试验应在一个能够及时恰当地处理急诊情况的地方进行，并选择适当的位置放置抢救车。

1. 应准备氧气、含服用硝酸甘油、阿司匹林和沙丁胺醇（定量吸入器或雾化器）。应有电话或其他求救方式。
2. 技术员应该具有进行初级生命支持的心肺复苏资质，高级生命支持资质也是需要的。相关医疗健康方面的培训、经验和资质（注册护士、注册呼吸治疗师、肺功能技师等等）也是需要的。据有相应资质的人员应该在需要时能及时赶到。
3. 并不是所有的试验都需要医师监护。申请该试验的医师或监查的实验室医师会决定在特殊试验时是否需要医师参加。
4. 正在接受持续氧疗的患者试验时需要接受平时水平的氧疗，或者服从医师或方案的指导。

需要立即停止6MWT的情况包括：(1)胸痛；(2)不能耐受的呼吸困难；(3) 下肢痉挛；(4)走路摇晃；(5)出汗；(6)面色苍白或灰白。

技术员必须接受培训以识别这些情况并正确处理。如果试验由于上述任何原因停止，根据具体情况或严重程度以及发生晕厥的风险大小患者应该坐下或平卧，技术员测量血压、脉率、氧饱和度，医师要对其进行评价，需要时应该给以氧疗。

**技术问题**

**试验条件**

6MWT应该在室内进行，沿着一条封闭的、长而直的平坦走廊进行，需要硬质地面。如果天气适宜，试验可以在室外进行。步行路线应30米长，因此需要100英尺的走廊。走廊的长度每3米处要有标记。折返处应有锥形标志（如同橙色交通锥标）。出发线为出发点和每个60米的终点，应该用明亮的颜色条带标于地面上。

**理论依据**

在短的走廊试验患者转身返回次数多，会减少六分钟步行的距离(6MWD)。多数研究采用了30米的走廊，也有一些采用20或50米走廊。近期一项多中心的研究发现步行路线长度从50到164英尺其结果没有显著差异，但在连续（椭圆形）道路上（均值92英尺）患者步行更远一点。

用踏车进行6MWT需要保存速度和全程监测,不建议使用。患者在踏车上不能自己控制速度。在关于严重肺疾病的研究中，6分钟内在踏车上步行的平均距离（患者自己调解速度）与在100英尺走廊上进行标准的6MWT相比平均缩短了14%。这种差别波动范围很大，在走廊上能步行1200英尺的患者在踏车上可以步行400-1300英尺。因此在踏车上试验的结果与走廊上试验的结果不能通用。

**所需设备**

1. 计时器(或秒表)
2. 圈数计数器
3. 两个小锥体用以标志转身返回点
4. 一把可以沿步行路线灵活移动的椅子
5. 放在剪贴板上的工作表
6. 氧气
7. 血压计
8. 电话
9. 除颤器

**患者的准备**

1. 穿着舒适.
2. 穿适于步行的鞋子.
3. 患者试验过程中应使用平时步行时使用的辅助物（拐杖、助步器等）.
4. 患者平时的治疗方案要继续。
5. 试验前饮食应清淡。
6. 试验前2个小时内患者应避免过度运动。

**测量过程**

1.为避免日内差异，重复试验应在每日大致相同的时间进行。试验前无需热身。

2.患者应在试验开始位置附近坐在椅子上休息至少10分钟。在此期间，检查是否存在禁忌症，测量脉搏、血压，确认衣服和鞋子适于试验。填写工作表的第一部分(见附件)。 3.可根据患者情况选择是否需要脉氧计。如果使用脉氧计，测量并记录基线心率和氧饱和度，按照说明书把信号调到最大同时把将运动伪影减小到最低，确定读数稳定。注意脉搏是否规律和脉氧计信号质量是否满意。

测量氧饱和度的理论依据：虽然步行的距离是初步测量结果，连续监测或许可以明确患者的病情改善，包括距离的增加或同样距离时症状的减轻。血氧饱和度在运动过程中不必持续监测。技术员也不必为了观察血氧饱和度而随患者一起步行。如果在步行时必须监测，则应使用轻质脉氧计（不超过2磅），使用电池并方便携带（可装在腰包里），这样患者就不需手持或因要保持它的稳定而影响步幅了。许多脉氧计运动伪影相当大而不能在步行时准确读数。

1. 患者站立并用Borg量表评价患者基线呼吸困难和疲劳情况（表2为Borg量表和使用说明）。

**表 2. BORG 量表**

| 0 正常 |
| --- |
| 0.5 非常非常轻微 (刚刚能察觉到) |
| 1 非常轻微 |
| 2 轻微 (轻度) |
| 3 中度 |
| 4 有些严重 |
| 5 严重（重度） |
| 6 |
| 7 非常严重 |
| 8 |
| 9 |
| 10 非常非常严重 (最大) |

注：6MWT开始前让患者阅读量表并询问患者：“请对照这个量表说出您的呼吸困难级别。”然后问：“请对照这个量表说出您疲劳的级别”。运动后重新评价呼吸困难和疲劳的级别，要提醒患者运动前所选的级别。

1. 将圈数计数器归零，计时器调到6分钟。准备好所有必需的设备（圈数计数器、计时器、剪贴板、Borg量表、工作表）并且放到出发点。
2. 按如下所示指导患者：

“这个试验的目标是在6分钟之内步行尽可能远的距离。您将在这个走廊上来会步行。6分钟的时间比较长，所以您在步行时要尽力去做。您可能会感到气喘吁吁或筋疲力尽，必要时可以放慢速度、停下来和休息。您可以靠着墙休息，但应争取尽快继续试验。”

“您要围绕锥体来回步行，在绕过锥体时不要犹豫停留。现在我给您做示范，请注意我转身时没有犹豫停留。”

“您自己要一圈一圈的走，步行时和绕过锥体时要轻快。”

“您准备好了吗？ 我将用计数器来记录您走完的圈数，每次您绕过出发线时都可以听到我按动它发出的嘀嗒声。记住目的是在6分钟内步行尽量远的距离，但不许跑或跳。”

“现在开始，或您准备完毕后开始。”

1. 让患者站在出发线上。试验过程中您也应该站在出发线附近，不要跟着患者步行。患者一开始走就开始计时。
2. 步行过程中不要跟任何人交谈，用平缓的语调和声音以及标准用语鼓励患者。要注意观察患者，不要走神而忘记计数圈数。每次患者回到出发线就要按动圈数计数器一次（或在工作表上标记圈数），并让患者看到它。计数时身体动作要夸张一点，如同比赛时使用秒表一样。

第一分钟过后，用平缓的语调告诉患者:“您做得很好，还有5分钟。”

当剩余4分钟时，告诉患者：“再接再厉，您还有4分钟。”

当剩余3分钟时，告诉患者：“很好，已经一半了。”

当剩余2分钟时，告诉患者：“加油，您只剩2分钟了。”

当只剩余1分钟时，告诉患者：“您做的很好，再走1分钟就结束了。”

不要使用其他鼓励性的语言（或肢体语言）。

如果患者试验过程中停住需要休息，告诉他：“您可以靠在墙上，觉得可以了就继续走。”不要停止计时器。如果患者在6分钟之前停下并拒绝再继续（或您判断他们不应该再继续）时，在工作表上记下步行距离、停止时间和过早停止的原因。

当还剩15秒时要对患者说：“过一会儿我说停下时您要立刻停在原地，我会过来。”

时间到了要说： “停！”然后走到患者身边。如果患者看上去很累要考虑给他们拿椅子。在他们停止的地方做一标识。

1. 试验后：记录Borg呼吸困难和疲劳水平，并问：“怎么样？怎么不能走得更远一点呢”
2. 如果使用了脉氧计，要测量血氧饱和度和脉率然后将其移开。
3. 记录步行的圈数
4. 记录最后未完成的一圈的距离，然后计算步行的总距离，记录在工作表上。
5. 对患者进行鼓励并提供饮用水。

**质量控制**

**影响因素**
 6MWD的影响因素很多（见表3）。由试验过程本身导致的差异应该尽量控制，要采用本指南的标准做法和质量控制程序。

表3. 6MWD的影响因素

| **减少6MWD的因素** |
| --- |
| 身材矮小 |
| 高龄 |
| 体重大 |
| 女性 |
| 认知障碍 |
| 走廊短（频繁转身） |
| 呼吸疾病（COPD、哮喘、囊性纤维化、间质性肺疾病） |
| 心血管疾病（心绞痛、心梗、心衰、中风、TIA、外周血管病、AAI） |
| 肌肉骨骼疾病（关节炎、踝、膝、髋关节损伤、肌肉萎缩等） |
| **增加6MWD的因素** |
| 身材高大（腿长） |
| 男性 |
| 强刺激 |
| 以前曾进行过该试验 |
| 试验前服药 |
| 运动性低氧血症患者吸氧 |

**练习试验**
 可以考虑练习试验，但并不需要在所有医疗机构均进行。如果进行练习试验，要等至少1小时再进行第二次试验，并且把这2次测试的最高值作为患者的基线值。

**理论依据**
 据报道一天中第二次六分钟步行试验的距离仅略有提高，范围0-17% (23,27,40,41,54,59)。一项470例重度COPD患者参加的多中心研究表明，患者进行2次6MWT，不在当日进行，第二次比第一次平均仅提高66英尺(5.8%)。

一周内进行2次试验后成绩常达到一个平台（未进行干预治疗）。培训的效果是提高协调性、找寻合适的步幅并且克服紧张情绪。这种练习或培训的效果在1月以后情况如何还没有研究报道，然而似乎在几个星期以后会逐渐减弱（不会持续存在）。

**技术员的培训和经验**
 进行6MWT的技术员应该接受标准方案的培训。他们在独立操作前需要在别人指导下进行几次试验，也应该完成心肺心肺复苏的培训。

**理论依据**

一项关于老年人的多中心研究发现在矫正了其他影响因素后，两个技术员操作的6分钟步行距离试验的距离比其他两个中心约低7%。

**鼓励**

试验过程中只能使用标准的鼓励用语（如前所述）。

**理论依据**
 鼓励能显著提高步行距离。测试时使用和不使用鼓励的重复性是相似的。一些研究在测试时每30秒、每分钟或每2分钟进行鼓励。我们采用每分钟使用标准用语进行鼓励。有的研究指导患者尽量快走。虽然这样获得的六分钟步行距离会长一些，但因其可导致过早的疲劳甚至使那些心脏病患者心脏负荷加重，因此我们不建议使用。

**氧气治疗**
 如果平时步行时需要氧气，并且计划进行该试验（在给予除氧疗以外的干预后），那在步行过程中需要给予和平时相同的给氧方式和流量。如果试验过程中症状加重需要提高氧流量，应该在工作表上记录，并且在解释六分钟步行距离的变化时要考虑到。给氧装置在报告上也要被注明：例如，患者携带液态氧或推/拉氧气瓶，氧气是脉冲式还是连续供给，或技术员带着氧气源走在患者后边（不建议）。给氧发生任何变化后至少10分钟后要测量脉搏和血氧饱和度。

**理论依据**
 对于COPD或间质性肺疾病患者，给氧可以提高6MWD(17,59,61)。在严重呼吸病患者中进行的研究表明，携带氧气袋（但没有用它供氧）可以使6MWD平均减少14%，但试验过程中用它供氧可以使6MWD提高20-36%。

**药物**
 要注意患者试验前使用的药物类型、剂量、服药间隔。

**理论依据**
 COPD患者在使用支气管扩张剂或心衰患者使用心血管药物后步行距离和呼吸困难程度显著改善。

**结果解释**

多数6MWTs会在干预前和干预后进行，在两次试验完成后首先要回答的问题是患者是否有显著的临床改善。有好的质量控制程序，由相同的技术员进行测试，经过1到2次练习试验，在保证这些条件的情况下6MWD的短期重复性很好。关于6MWD的变化用以下哪种方式表达对临床的意义最大目前尚无定论(1)绝对值(2)百分比, 或(3)变化占预计值的百分比。在没有进一步的研究之前我们建议使用绝对值（如患者6MWD增加了50米）。

患者6MWD的提高不如临床改善明显。在一项112例（一半为女性）稳定期重度COPD患者研究中，与显著的临床变化相关的最小6MWD变化均值为54米(95%可信区间，37-71米)(64)。这项研究建议对于COPD的患者，一项能使6MWD增加超过70米的干预是有显著性意义的（可信性为95%）。一项45例老年心衰患者的观察研究表明与临床病情显著变化相关的6MWD平均变化为43米。6MWD反映心衰症状的恶化比改善更敏感。

**干预后6MWD的变化的平均值**
 在一项研究中COPD或间质性肺疾病的患者在吸氧(6 L/min)的情况下6MWD平均增加约83米(36%)。一项国际COPD研究表明使用吸入激素治疗可使6MWD平均增加33米(8%)。关于运动效应和隔肌力量训练的研究发现可使6MWD增加50米(20%)。非常严重的COPD患者进行肺减容术可使6MWD平均增加55米(20%)。

一项近期的研究表明心脏病患者进行心脏康复可以使6MWD增加170米(15%)。在25例老年心衰患者中ACEI(卡托普利 50mg/每日)可以使6MWD平均增加64米（39%），而安慰剂组仅平均增加8%。

**功能状态测量结果解释**

目前尚没有健康人群使用标准6MWT方法的数据结果。在一项研究中，6MWD的中位数117名健康男性约为580米，173名健康女性约为500米。另一项研究报道51例健康老人的平均6MWD为630米。研究人群的不同、鼓励方式和频率的不同、走廊的长度的不同和试验前练习次数的差异均可导致健康人平均6MWD的不同。年龄、身高、体重和性别是健康人6MWD的独立影响因素，因此，这些因素在解释结果时应该考虑。我们鼓励研究者使用标准程序进行6MWT以得到正常人群的数据结果。

6MWD减低不具有特异性和诊断性。当6MWD减低时应进行全面检查以明确原因。下列相关试验可供参考：肺功能、心功能、踝-肱比值、肌力、营养状态、骨功能和认知功能。

**结论**
 6MWT是一项检测功能代偿能力方法，适用于至少中等程度受损的患者。该试验广泛应用于心肺疾病治疗干预前后的临床评价。本指南提供了一个进行6MWT的标准方法。制定该指南的委员会希望它能鼓励大家对6MWT进行更深入的研究并使不同的研究之间具有可比性。

**附件**

| \| 下列项目应在6MWT工作表和报告上详细填写 \| \| \| \| --- \| --- \| --- \| \| 圈数计数器:__ __ __ __ ____ __ __ __ __ __ __ __ __ __  患者姓名: ____________________ 患者 ID# ___________  试验编号______ 技术员 ID: _________ 日期: __________  性别: M F 年龄: ____ 民族: ____ 身高: ___ft ____in, ____ meters  体重: ______ lbs, _____kg 血压: _____ / _____  试验前用药 (剂量和时间): __________________  试验时是否需要氧气: 否/是, 流量 ______ L/min, 方式 _____ \| \| \| \|  \| 基线 \| 试验结束 \| \| 时间 \| ___:___ \| ___:___ \| \| 心率 \| _____ \| _____ \| \| 呼吸困难 \| ____ \| ____ (Borg 量表) \| \| 疲倦 \| ____ \| ____ (Borg 量表) \| \| SpO_2_ \| ____ % \| ____% \| \| 试验是否提前结束? 否 是, 原因: _______________  试验结束时的其他症状: 心绞痛 头晕 臀、大腿或小腿痛  圈数: ____ (x60米) + 最后未完成的一圈: _____ 米 = 6分钟步行总距离: ______ 米  预计值: _____米 占预计值百分比: _____%  技术员注解:  结论(包括与干预前6MWD的比较): \| \| \| |
| --- | --- | --- | --- | --- | --- | --- | --- | --- | --- | --- | --- | --- | --- | --- | --- | --- | --- | --- | --- | --- | --- | --- | --- | --- | --- | --- | --- |

# 附录3：mMRC评分

| **mMRC分级** | **呼吸困难严重程度** |
| --- | --- |
| 0 | 我仅在费力运动时出现呼吸困难 |
| 1 | 我平地快步行走或步行爬小坡时出现气短 |
| 2 | 我由于气短，平地行时比同龄人慢或者需要停下来休息 |
| 3 | 我在行走100m左右或数分钟后需要停下来休息 |
| 4 | 我因严重呼吸困难以致不能离开家，或在穿衣服、脱衣时出现呼吸困难 |

**方案签字页**

我作为参与研究的医生/统计分析人员，已经阅读过这项研究的方案。

我已经与研究负责人充分讨论了这项研究的目的和本方案的内容。

我同意根据本方案进行研究，并遵守其要求，遵守伦理规范，并在药物临床研究质量管理规范（GCP）指导下开展本项临床研究。

我同意对本研究方案的内容保密，不会透露给第三方，且方案的内容仅用于进行这项研究。

我理解，如果本项研究在任何时间以无论什么原因作出提前终止或暂停这项研究的决定，都将以书面形式通知我。同样，如果我决定退出执行这项研究，也会以书面形式立即通知本研究负责单位及主要研究者。

| 研究中心： |  |
| --- | --- |
| 研究者签名： |  |
| 地址： |  |
| 电话： |  |
| 日期： | 年 月 日 |
